# Mode of Infection of *Metarhizium* spp. Fungus and Their Potential as Biological Control Agents

**DOI:** 10.3390/jof3020030

**Published:** 2017-06-07

**Authors:** Kimberly Moon San Aw, Seow Mun Hue

**Affiliations:** School of Science, Monash University Malaysia, Jalan Lagoon Selatan, Bandar Sunway, 47500 Subang Jaya, Malaysia; kimaw94@gmail.com

**Keywords:** biopesticide, *Metarhizium anisopliae*, *Metarhizium acridum*, biological vectors, agricultural pests, mechanism of infection

## Abstract

Chemical insecticides have been commonly used to control agricultural pests, termites, and biological vectors such as mosquitoes and ticks. However, the harmful impacts of toxic chemical insecticides on the environment, the development of resistance in pests and vectors towards chemical insecticides, and public concern have driven extensive research for alternatives, especially biological control agents such as fungus and bacteria. In this review, the mode of infection of *Metarhizium* fungus on both terrestrial and aquatic insect larvae and how these interactions have been widely employed will be outlined. The potential uses of *Metarhizium anisopliae* and *Metarhizium acridum* biological control agents and molecular approaches to increase their virulence will be discussed.

## 1. Introduction

Pests such as locusts, grasshoppers, termites, and cattle ticks have caused huge economic and agricultural losses in many parts of the world such as China, Japan, Australia, Malaysia, Africa, Brazil, and Mexico [1,2,3,4,5,6,7,8]. Vectors of malaria, dengue, and *Bancroftian filariasis*, which are *Aedes* spp., *Anopheles* spp., and *Culex* spp. respectively, have been responsible for hospitalization and death annually [9,10]. To eliminate these pests and vectors, chemical insecticides have been commonly used as the solution. However, the application of chemical insecticides has entailed ground water contamination and detrimental effects on the natural enemies of pests [5,6]. Besides, the development of resistance of these agricultural pests, termites, and biological vectors have driven intensive research in substituting chemical insecticides with biological control agents such as bacteria, viruses, nematodes, and fungus [5,6,8,9,10,11,12,13,14].

Entomopathogenic fungus such as *Metarhizium* spp. has been widely studied because of its narrow host range, safety, environmental friendliness, and ease of mass production [10,15,16,17,18]. The *Metarhizum* genus is distributed globally from the artic to the tropics. It belongs to the class Hyphomycetes, which reproduce by spores known as conidia [19]. The Metarhizium genus was originally comprised of four varieties, which were *M. anisopliae*, *M. taii*, *M. pingshaense*, and *M. guizhouense*. However, in 2009, Bischoff, Rehner, and Humber reclassified the Metarhizium genus into nine species, which are *M. anisopliae*, *M. acridum*, *M. guizhouense*, *M. pingshaense*, *M. lepidiotae* and *M. majus*, *M. robertsii*, *M. brunneum*, and *M. globosum* [20].

The Metarhizium genus comprises mostly entomopathogenic fungi: some are generalists, while some are specialists [21]. The enthomopathogenic fungus *M. anisopliae* is a generalist and is known to infect insects from more than seven orders, while *M. acridum* is a specialist as it only infects insects from the Acrididae family [22]. 

Globally, countries have used *M. anisopliae* and *M. acridum* as an active ingredient in the development of mycosecticides and mycoacaricides, respectively, to target pests in their countries [23]. An example is BIO 1020, a mycoinsecticide developed by Bayer, Germany, which contains *M. anisopliae* as an active ingredient to target pests from the Coleoptera species; another example is Green Guard^®^; a mycoacaricide developed by Becker Underwood Inc. (Ames, IA, United States) that contains *M. acridum* as an active ingredient to target pests from the Orthoptera family.

There are extensive studies on the general mode of infection of *Metarhizium* spp. on terrestrial arthropods. These studies are crucial in contributing to the understanding of the precise mechanism of infection which allows the identification of the genes responsible for its virulence and pathogenicity, as well as the enzymes and toxins produced. However, few studies have compared the genes responsible for the difference in the host specificity between *M. acridum* and *M. anisopliae* and the effects on their virulence and pathogenicity [21,24].

Pests such as termites jeopardise buildings and monuments, and locusts and grasshoppers damage crops, while biological vectors such as mosquitoes and ticks, which caused a worldwide epidemic, have forced governments to spend huge sums of money from approximately USD 400 million to USD 2 billion solely to control these pests and biological vectors [6,25,26]. Hence, it has been of great interest to conduct research on cheaper and more environmental friendly methods to control these pests.

In this review, the interactions between two fungi from the *Metarhizum* genus, which are *M. anisopliae* and *M. acridum*, and their insect hosts, will be evaluated. The potential uses of *M. anisopliae* to control termites, mosquitoes (*Aedes* spp., *Anopheles* spp. and *Culex* spp.), and cattle ticks, and the potential use of *M. acridum* to control locusts and grasshoppers, will be discussed.

## 2. Mode of Infection of *Metarhizium* spp. with Their Hosts

The general mode of infection of *Metarhizium* spp. comprises six stages in the following order: adhesion, germination, appressorium formation, penetration, colonization of haemolymph, and extrusion and sporulation, which can also be found in other entomopathogenic fungi. However, *Metarhizium* spp. has genes that are specific to each species in their mode of infection, such as the *Mad1* and *Hog1* kinase genes for the germination stage in *M. anisopliae* and *M. acridum*, respectively.

### 2.1. Adhesion

For *M. anisopliae*, the adhesion stage is the most crucial stage for successfully infecting the host [27]. Conidia, which are the asexual spores of *Metarhizium* spp., adhere to the waxy epicuticle of their host through a combination of passive hydrophobic forces, electrostatic forces, and protein interactions between the conidia and the epicuticle. The outer layer of cells on the conidia, also known as rodlets, contains hydrophobins, which are proteins that facilitate the adhesion of conidia to the hydrophobic epicuticle [28,29,30]. The attachment of conidia onto the cuticle is affected by factors such as the topography and chemical composition of the host’s cuticle, the hydrophobicity of the host’s surface, the feeding habits of the host, and the environmental conditions [28,31,32]. As for *M. acridum*, to date, no research has been published on the enzymes responsible for adhering the fungus to its host or whether the enzymes are similar or different to those of *M. anisopliae*.

### 2.2. Germination

After the adhesion of conidia to the host cuticle, conidia germination in *M. anisopliae* is initiated by the presence of exogenous carbon and nitrogen sources, whereby the latter is preferentially used [33]. Trehalase, which utilizes trehalose commonly found in the host haemolymph, could be observed during early germination. The activity of trehalase was presumed to supply glucose for energy production [34,35]. After germination, the spores swelled, producing germ tubes which differentiate into appressorium [36]. Hydrophobins are replaced by adhesins, namely *Mad1* and *Mad2*, specific to *M. anispliae* and more firmly attach the fungus onto the cuticle, enabling conidial germination and subsequently, appressorium formation [29,37].

Similar to *M. anisopliae*, *M. acridum*, which is acridid specific, require simple polar compounds such as fatty acids, glucose, amino acids, fatty acid esters, and peptides present on the surface of the wings of acridids to promote fungus germination. Unlike *M. anisopliae*, whereby non-polar lipids on the surface of the cuticle could stimulate germination immediately, *M. acridum* has to use up the polar compounds available before it can utilize non-polar lipids on the cuticle [35,38]. The difference between *M. anisopliae* and *M. acridum* in stimulating germination is that mixtures comprising different types of hydrocarbons from insects promote better germination in *M. anisopliae* compared to *M. acridum*, which could only germinate better in the presence of a single type of hydrocarbon [38]. In addition, the *MaHog1* gene found only in *M. acridum* encodes for Hog1 kinase which contributes to conidia germination [39].

### 2.3. Appressorium Formation

The *ODC1* gene and *Mpl1* gene specific to *M. anisopliae* were demonstrated to be responsible in appressorium formation. The *ODC1* gene, which encodes for ornithine decarboxylase, was upregulated during conidia germination and germ tube differentiation to form appressorium, while the *Mpl1* gene which encodes for MPL1, regulates lipid homeostasis and appressorium differentiation [40,41]. Besides, protein kinase A was demonstrated to be involved in ergosterol biosynthesis, which regulates the permeability of glycerol under hypoosomotic conditions besides maintaining turgor pressure in appressorium [42]. This turgor pressure generates the mechanical pressure required for the penetration of the host cuticle [29,36]. A thin layer of mucilage is also secreted by appressorium to consolidate the attachment of the fungus to the cuticle [28,29,32,43].

Similar to *M. anisopliae*, Fus3/Kss1-type mitogen-activated protein kinase (MAPK), a family of proteins commonly found in other entomopathogenic fungus, was encoded by the gene *MaMk1*, specific to *M. acridum*, and was found to be involved in regulating the expression of *Mad1* genes which are responsible for adherence and the *Mpl* gene which regulates lipid metabolism and appressorium formation [44]. Besides, *MaMk1* also encodes for an extracellular signal-regulated kinase, which is also involved in appressorium formation. Tetraspanins encoded by the *MaPls1* gene were also demonstrated to be involved in pathways affecting the turgor pressure of appressorium, that in turn influences appressorium formation and the delayed germination of *M. acridum* [45].

### 2.4. Penetration

The penetration stage in *M. anisopliae* involves the secretion of proteins such as subtilisins, trypsins, chymotrypsins, and carboxypeptidases, which digest the protein rich procuticle of arthropods [46]. The types and amount of proteins produced by *M. anisopliae* are found to be specific for each host, explaining its ability to infect many different hosts. Trypsins are host specific as they were found to be produced only in certain hosts such as cockroaches and beetles [31]. Subtilisin proteases such as Pr1, are cuticle degrading enzymes which penetrate the cuticle by hydrolyzing the cuticular proteins during nutrient deprived conditions. In the presence of exogenous carbon and nitrogen sources, its production is repressed [27,29]. Chitinases, which work synergistically with proteases to digest host cuticles, were also produced. In different hosts, different isoforms of chitinases are produced [29]. Lipases, which may be present on the surface of conidia, also improve the adhesion of the conidia to the host by enhancing the hydrophobic interactions between the conidia and the host through the release of free fatty acids through its lipolytic activity [34,47]. Collectively, chitinases, proteases, and lipases degrade the cuticle, respectively, to enable the successful penetration and utilization of nutrients in the haemocoel of the host for efficacious infection [34].

Similar to *M. anisopliae*, *M. acridum* also contains genes that encode for cuticle degrading enzymes such as trypsins and subtilisin proteases were also produced. In addition, aspartyl proteases, glycoside hydrolases, and lipases are also present on the host cuticle [22]. Pr1, which is a protease, was suggested to act synergistically with aminopeptidases to hydrolyse the cuticle proteins. During the penetration stage, aminopeptidases and mucilage were produced in the mature appressorium and on the wings of locusts, respectively [48]. Jin et al. [39] also found that the *MaHog1* gene, which encodes for Hog1 kinase, regulates the penetration and extrusion of *M. acridum* on its host. The *Chi2* gene, which encodes for an endochitinase, was also crucial for the penetration of the host cuticle by reducing the lethal time that is the time required to kill the host [49]. Luo et al. [45] found that the *MaPls1* gene encodes for tetraspanins that regulate the turgor pressure of appressorium and the expression of cuticle-degrading enzymes, particularly enzymes responsible for the degradation of protein and chitin. The disruption of this gene resulted in the inability of the hyphae to reach the haemocoel of the host and differentiate to form appressorium. Tetraspanins were also reported to regulate the expression of *ApsA* and kinesin, which are responsible for the structure and migration of the cytoskeleton. They also contribute to sensing the host-specific environment and respond by commencing germination using nutrients on the cuticle of the host. Furthermore, tetraspanins encoded by *MaPls1* also crosstalk with calmodulin-dependent signalling pathways, which are responsible for regulating the growth of fungus in the host environment, appressorium formation, and the activity of GTPases, which in turn regulate cellular activities. 

### 2.5. Colonization of the Haemolymph

In *M. anisopliae*, destruxins, especially destruxins A and E which are more insecticidal, are synthesised to repress the cellular and humoral immune response in the host. This is done by enabling *M. anisopliae* spores encapsulated by the host haemocytes to escape. Destruxin biosynthesis was proposed to be regulated by the dtxS1 gene cluster commonly found in *Metarhizium* generalists’ genome such as *M. anisopliae*, but not in the *M. acridum* genome [50]. Evasion proteins such as *Mcl1* are also produced to allow the fungus to evade the host immune system [46]. 

To protect the conidia against reactive oxygen species formed by ultraviolet radiation and heat in the environment, catalase and peroxidases are also present on the conidia surface [34]. Besides, trehalases are also produced to convert trehalose commonly found in the haemolymph of the host into glucose needed for energy production [33,34]. *Mad1* proteins initiate the expression of genes involved in the cell cycle, enabling the rapid multiplication and differentiation of hyphae in the haemolymph of the host. These proteins orientate the cytoskeleton and regulate cytokinesis in the cell cycle [37,46].

Unlike *M. anisopliae*, toxin production is limited in *M. acridum.* The virulence of *M. acridum* relies mainly on its proliferation inside the host haemocoel [51]. Similar to *M. anisopliae*, *M. acridum* utilizes trehalose, which is the major blood sugar present in the haemolymph of an insect. Trehalase encoded by *ATM1* in *M. acridum* was found to reduce fecundity, and impair homeostasis and insect physiology, besides interfering with its locomotive behaviour, particularly its flight movements [52,53]. 

An adenylate cyclase gene known as *MaAC* was found to regulate the stress tolerance of *M. acridum* to both the environment and the haemolymph of an insect such as hydrogen peroxide, ultraviolet radiation, thermal stress, osmotic stress, and locust fever. *MaAC* was also involved in cAMP synthesis, which regulates morphogenesis and the cellular processes of the host [54]. Similar to the *MaAC* gene, the *Hog1* gene encodes for Hog1 kinase, which reduces the sensitivity of the fungus to environmental stress such as high temperature, hyperosmotic stress, and oxidative stress. From the genome of *M. acridum*, Gao et al. [21] found that *M. acridum* contains cytochrome P504s genes, which also function to degrade phenylacetate, an antimicrobial compound in the host.

### 2.6. Extrusion and Sporulation

During sporulation, the hyphae extrudes the host cuticle to the outer environment. *M. anisopliae* forms a denser network and green spores on the cadaver of the infected host [55,56].

In comparison to *M. anisopliae*, which forms green spores on the cadaver, red sporulation was observed on the cadaver of locusts and grasshoppers for *M. acridum* [57]. In *M. acridum*, Hog1 kinase was responsible for the extrusion of hyphae from the cuticle of the infected host [39]. The filamentous growth and conidiation of *M. acridum* was observed to be regulated by the catalytic subunit A of calcineurin, a Ca^2+^ calmodulin-dependent serine-threonine protein phosphatase [58]. This catalytic subunit also regulates chitin synthesis and β-1,3-glucan, which are major components of the cell wall of *M. acridum*. The deletion of this subunit was found to lead to thinner cell walls. β-1,3-glucan synthase encoded by the *MaFKS* gene was also responsible for mycelium growth and conidia production during the sporulation stage, besides regulating hyperosmotic pressure tolerance which contributes to cell wall integrity [59]. Calcineurine also contributes to the regulation of calcium transport-related genes that modulate calcium influx and homeostasis in fungi. Calcium is crucial in signal communication between the interior and exterior of the cell. Conidia production and blastospores were also observed to be reduced during the downregulation of the *Aba* gene, which is crucial for phialides differentiation and functionality [58]. 

Collectively, calcineurine downregulates the protein kinase A, small G protein, cAMP-PKA, and MAPK signalling pathways, which are important in the regulation of growth, conidiation, stress tolerance, and cell wall integrity.

### 2.7. Summary

**Figure 1:**
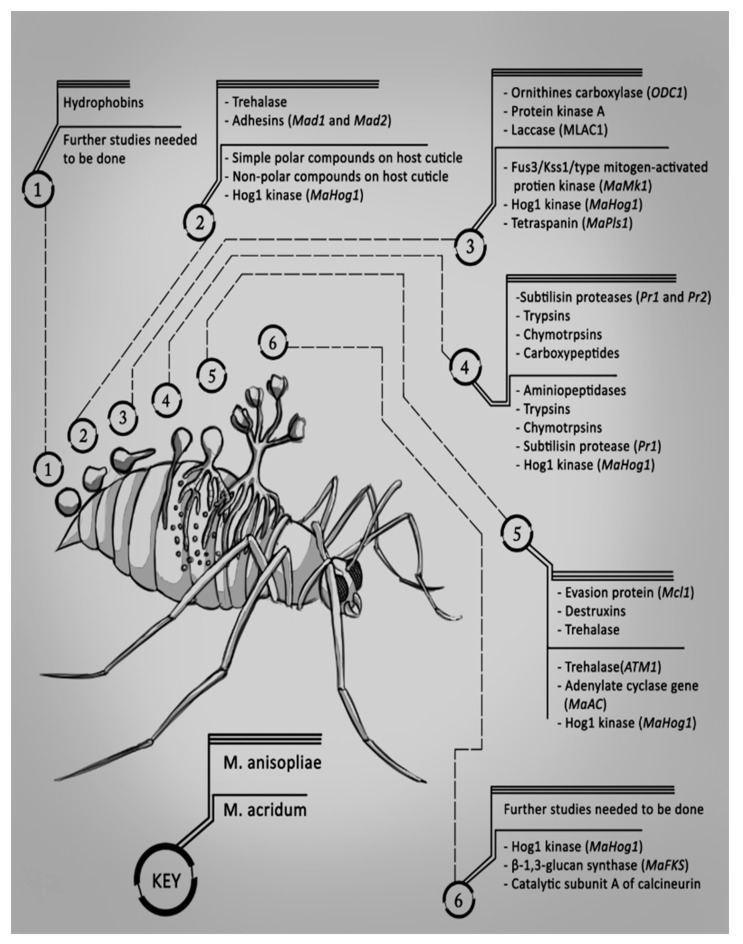
The genes and the proteins encoded produced by *M. anisopliae* and *M. acridum* during each stage of infection.

A summary of the proteins produced by *M. anisopliae* and *M. acridum* during the six stages of infection is shown in Figure 1. The genes which encode for the production of these proteins are also included in brackets beside the proteins.

## 3. Mode of Infection of *Metarhizium* spp. in Aquatic Insect Larvae

Studies have discovered that *M. anisopliae* displays a significant efficacy against aquatic hosts such as larvae of *Spodoptera litura* [60,61] and *Aedes aegypti* [29,31,62]. Therefore, different studies have suggested different mechanisms of infection of *M. anisopliae* in the aquatic hosts. However, the exact mode of infection of *M. anisopliae* is still unclear.

Similar to a terrestrial host, the expression of proteinases by *Pr* genes and adhesins by *Mad* genes is upregulated in the presence of aquatic larvae, signifying the recognition of *M. anisopliae* by its host [29,32]. However, unlike its interactions with terrestrial hosts, Butt et al. [29] and Greenfield et al. [32] noted that the conidia were unable to attach firmly onto the aquatic larvae, resulting in the inability of *M. anisopliae* to penetrate the host cuticle to infect and reproduce. Greenfield et al. [31] also found that long-chain hydrocarbons present on the cuticle of the terrestrial host, but absent in *Aedes* larvae, are most likely responsible for the failure of the attachment of conidia to aquatic larvae. This indicates that the mode of infection of *M. anisopliae* is not via the penetration of the host cuticle due to the absence of three long-chain hydrocarbons necessary for the attachment of adhesins produced by *Mad* genes to infect the host.

The most likely mode of infection of *M. anisopliae* is through the ingestion of spores by the larvae [18,19,29,32]. Microscopic observations revealing the ingestion of conidia by larvae exposed to *M. anisopliae* and the presence of conidia in the faecal pellents of mosquito larvae demonstrated that conidia had been ingested. Further researches are needed to determine the precise mode of infection of *M. anisopliae* in the larvae [18].

Although *M. anisopliae* did not manage to penetrate the cuticle to infect its host, the larval mortality was still significant. Three hypotheses for the mechanism of infection of *M. anisopliae* have been proposed. The first hypothesis is that the conidia germinated inside the larvae produce hyphae, the which may block the respiratory siphon, resulting in suffocation and death of larvae [10,62]. The second hypothesis is that destruxins were the cause of larval mortality, while the third hypothesis is that the presence of conidia triggered the production of extracellular proteases in the midgut of larvae [29].

In response to the first hypothesis, Butt et al. [29] reported that heat killed conidia were found not to cause significant larval mortality compared to the control without conidia. However, Greenfield et al. [10] found that high concentrations of heat killed spores were able to cause significant larval mortality. They hypothesised that there are other factors which may contribute to larval mortality besides the proteases produced by viable conidia which were reported to cause significant larval mortality [10]. Future research could be conducted on how high dosages of heat killed conidia are capable of causing significant larval mortality. 

For the second hypothesis, destruxins were reported to trigger oxidative stress in larvae, leading to larval mortality [60,61]. An increase in lipoxygenase, superoxide dismutase, catalase, peroxidase, ascorbate peroxidase activity, and lipid peroxidation levels in the larvae of *S. littura* treated with *M. anisopliae* were also found to be different in larvae before and after infection by *M. anisopliae* [62]. Oxidative stress in *S. littura* was indicated via the increased levels of protein carbonyls and free radicals. Damage of the salivary gland and epithelial cells in the midgut of *S. littura* was also observed.

However, destruxins were not detected by Butt et al. [29], leading to the third hypothesis that extracellular proteases (*Pr1* and *Pr2*) were produced when conidia passed through the mid gut. Butt et al. [29] treated the conidia with egg white, which contains protease inhibitors. Butt et al. [29] showed that larval mortality is significantly higher in the presence of proteases, which upregulates the expression of genes that increase the activity of caspases. Caspases induce apoptosis reaching beyond the threshold of dead cells. Therefore, further studies have to be done to determine whether only one hypothesis is true or whether a combination of the occurrence of the three hypotheses is necessary to cause significant larval mortality. 

Another challenge in utilizing *M. anisopliae* conidia as biopesticides for targeting aquatic insect larvae is the ability to increase the encounter between *M. anisopliae* and target aquatic insect larvae in still waters, as the property of *M. anisopliae* being an aerial conidia is that it is only effective when it comes into contact with aquatic hosts. A better understanding of the mode of infection of *M. anisopliae* is crucial for the development of *M. anisopliae* as an effective biopesticide.

## 4. Molecular Approaches to Increase the Virulence and Efficacy of *Metarhizium* spp. as a Biological Control Agent

The efforts to increase the virulence of *Metarhizium* fungus have been done by either overexpressing genes which are responsible for the pathogenicity of the fungus or through genetic manipulation, by inserting genes from scorpions or spiders which produce insect-specific neurotoxins to increase the virulence of the fungus [63].

Morales Hernandez et al. [64] overexpressed the *cat1* gene in *M. anisopliae* which increases the activity of catalase, contributing to a higher tolerance towards exogenous hydrogen peroxide. Transgenic conidia of *M. anisopliae* were also found to accelerate conidia germination, contributing to an increased virulence of *M. anisopliae*.

Wang and St. Leger [65] inserted the *AaIT* gene, which encodes for an insect-specific neurotoxin from a buthid scorpion *Androctonus australis* in *M. anisopliae.* They reported that the amount of genetically engineered conidia required to provide the same level of mortality with the wild type *M. anisopliae* was nine-fold lower. The median lethal time of *M. anisopliae* on *A. aegypti* was also significantly reduced. *A. aegypti* infected with this neurotoxin was found to have spasmic legs and affected wing movements.

Fang et al. [66] genetically engineered the *M. anisopliae* to express a salivary gland and midgut peptide (SM1) which attach to the surface of the salivary gland of *Anopheles* spp., effectively blocking the entry of sporozoites of *Plasmodium falciparum* which is the causal agent of malaria. A synthetic gene that encodes for the production of an antimicrobial scorpine which expresses eight repeats of the SM1 peptide was also inserted into the genome of *M. anisopliae*. This scorpine not only attached to the salivary gland of the mosquitoes, but also reduced the sporozoite density in the haemolymph, thus reducing the transmission of malaria. 

Zhang et al. [67] genetically engineered *M. anisopliae* by inserting a gene from *Bacillus thuringiensis* which encodes for an insect midgut-specific toxin Vip3Aal. The expression of this toxin enables *M. anisopliae* to acquire the ability to infect *Spodoptera litura* larvae, which is usually resistant to *M. anisopliae*, through the ingestion of conidia instead of the usual mode of infection that occurs through penetration of the cuticle.

The overexpression of the *ATM1* gene on *M. acridum* was performed to increase the production of acid trehalase. This increased the activity of acid trehalase, enabling the breakdown of more trehalose in the haemolymph of the host into glucose, thus producing more energy for *M. acridum* and subsequently decreasing the time taken for fungus to grow and colonise, increasing the efficacy and virulence of *M. acridum* [53].

Fang et al. [15] genetically engineered four *M. acridum* strains, whereby each strain expressed one insect specific neurotoxin. The genes introduced into the *M. acridum* strains are genes which encode functions as a blocker of voltage gated sodium, calcium, and calcium activated potassium channels. The last gene encodes for a hybrid toxin which interferes with both calcium and potassium channels in the host. All *M. acridum* strains have a significantly higher virulence than the natural *M. acridum* strain, with the *M. acridum* strain expressing the hybrid toxin with the highest virulence measured in terms of effective conidial doses, median lethal time, and the food consumption of the host.

The insertion of the *LqhIT2* gene, which encodes for a neurotoxin from the venom of the scorpion *Leiurus quinquestriatus hebraeus*, into the genome of *M. acridum* was found to increase fungal virulence. *M. acridum* expressing the *LqhIT2* gene grew faster and reduced the median lethal time (LT_50_) and median lethal dose (LC_50_) by 30.3% and 22.6-fold, respectively, compared to the wild type. The neurotoxin produced was reported to have no effect on the cuticle penetration and germination stage. Peng et al. [51] reported that the expression of the *LqhIT2* neurotoxin may have suppressed the host immunity, resulting in the accelerated growth of conidia in the haemolymph of host. 

Peng and Xia [63] inserted a gene from a species of scorpion, *Buthotus judaicus*, which encodes for the insect-selective neurotoxin BjαIT to determine whether this gene increased the virulence of *M. acridum*. The neurotoxin was found to speed up the growth of *M. acridum*, which decreases the mean lethal time and dose time needed to kill the host, thus increasing its virulence. The engineered *M. acridum* is environmentally safe as it did not affect non-target insects and its safety can be improved by using a tissue-specific promoter such as the *Mcl1* promoter. However, the conidial yield of the engineered *M. acridum* was reduced, indicating that the residual effect of *M. acridum* will be lower compared to the wild type.

The efforts to increase the virulence of *Metarhizium* spp. could be improved by overexpressing several targeted genes such as the *cat1* gene and *ATM1* gene in *M. anisopliae* and *M. acridum*, respectively. Fang et al. [15] and Peng and Xia [63] have successfully inserted genes that are able to increase the virulence of *M. acridum*; however, further studies could be conducted investigating how to prevent the decline in conidial production. Future studies should also be conducted on the impacts of transgenic fungus on the environment and non-target organisms.

## 5. Mass Production of *Metarhizium anisopliae* and *Metarhizium acridum*

Studies have demonstrated that a different medium could affect the conidial morphology and conidia germination rate, which could subsequently affect the virulence of *M. acridum* [68]. PikKheng et al. [4] found that Sabaroud Dextrose Agar with 1% yeast extract (SDAY) yielded the *M. anisopliae* with the thickest conidia and the greatest colony growth with local isolate. In a separate study, Jenkins medium yielded the most *M. anisopliae* with 100% mortality on the fourth day with 1 × 10^9^ conidia/mL [67]. 

For the conidia production, it was found that the type of culture medium used to produce *M. anisopliae* is capable of affecting its virulence. A study found that the virulence of the different strains was influenced by different medium cultures [69]. This may be due to the different nutrients required by different strains of *M. anisopliae* that are genetically different. In addition, Maldonado-Blanco et al. [69] also found that the mortality of *Aedes* spp. increases with an increasing incubation time with the spores. For the mass production of *M. anisopliae*, the strain of *M. anisopliae* used should be taken into consideration as different strains of *M. anisopliae* have different phenotypic plasticity which may affect the results for the determination of which medium produces the most virulent *M. anisopliae* [4].

The type of culture medium used to produce *M. acridum* also affects the characteristics of conidia, such as their virulence [70]. Rangel et al. [70] also found that the conidia of *M. acridum* produced on potato dextrose agar supplemented with 1 g L^−1^ yeast extract (PDAY) germinated faster and had a higher UV-B tolerance compared to conidia produced on insect cuticles. Rangel et al. [70] hypothesised that conidia of *M. acridum* germinated on potato dextrose agar may have accumulated more endogenous nutrients, allowing them to germinate faster. This finding is crucial when mass producing *M. acridum* for a biological pesticide as it is advantageous to use a culture medium to produce a virulent and high amount of conidia of *M. acridum* [70].

## 6. Conidia Formulation for *Metarhizium anisopliae* and *Metarhizium acridum*

The effect of oil formulation, oil-in-water emulsion formulation, and water formulation of *M. anisopliae* conidia has been tested and compared for different hosts. Oil formulated conidia caused higher mortality in hosts compared to water formulated conidia [3,8,25,71,72,73,74,75,76,77,78]. Besides, oil formulated conidia were found to provide thermotolerant protection to the conidia and remain effective even under low humidity conditions. The efficacy of oil formulated conidia was proposed to be due to its ability to protect conidia from heat, sunlight, and humidity, besides enhancing the attachment of conidia onto the host cuticle [76,79]. Besides, egg production in engorged female ticks and larval eclosion were reduced when the hosts were infected with *M. anisopliae* conidia [73,76]. Further studies should be done on how oil reduces larval eclosion, focusing on whether oil promotes the germination of conidia or reduces the amount of oxygen needed for a mosquito to survive. Studies using a scanning electron microscope, such as the experiment done by Leemon et al. [80], which observe the development of germ tubes and the formation of appresorium could be used to compare and determine whether oil formulated conidia exhibit a greater attachment to the cuticle compared to aqueous formulated conidia. 

Although oil formulated conidia were found to be efficacious under a low humidity and high temperature, Lobo et al. [79] found that *A. aegypti* decreased the tendency to oviposit in breeding sites with a high oil concentration. This could potentially reduce the contact between the fungal propagules and the host. Lobo et al. [79] proposed that ovitraps containing moisture which attracts female mosquitoes to oviposit may help to overcome this problem. Besides, oil formulation may prevent conidia from obtaining the water needed for germination, especially during a drought, where termites are at their most active.

The type of oil formulation was demonstrated to affect the viability of conidia whereby *M. anisopliae* conidia suspended in pure mineral oil provided a greater thermoprotection compared to canola oil formulated conidia [81]. The components in a different formulation may differ in their ability to confer conidia protection. 

Most studies have studied the effect of a single variable, such as heat, on the efficacy of conidia and the effect of the period of exposure on the efficacy of conidia. Future studies could be conducted to find a way to combine the effects of heat and humidity on oil formulated conidia. Paixão et al. [82] also suggested that pH could affect the efficacy of *M. anisopliae* conidia by changing the fatty acid composition on the host cuticle. Future studies could manipulate the pH of the oil formulated conidia and determine whether pH could retain the virulence of the conidia for longer periods of time.

For the formulation of the conidia of *M. acridum*, Peng and Xia [12] reported that emulsion formulation is better than oil formulation as emulsion formulated conidia persist for longer in the environment, especially when the humidity in the environment is low, besides having a similar thermotolerance and UV-B tolerance to oil formulated conidia. This finding is contradictory to the findings from Hussain et al. [71] and Sousa et al. [73]. They theorised that emulsion formulated conidia at a low humidity remained virulent and persisted for longer because the water necessary for conidia germination is available, allowing a more rapid penetration of the host cuticle. Further studies need to be done on whether water is a necessity for germination, since studies using *M. anisopliae* found that oil-formulated conidia could grow on mosquito eggs [73]. The oil in the emulsion also protects conidia from environmental conditions such as a high temperature and solar radiation [11]. However, for the storage of the conidia of *M. acridum*, dry spore formulation remained virulent for longer periods of time during storage compared to oil formulated conidia [83].

## 7. *Metarhizium anisopliae* as a Biological Control

### 7.1. Biological Control of Termites

Termites are important pests of buildings and historical monuments due to their ability to form colonies in walls and damage wood in the process [6,13]. The costs required to control termites were estimated to be around $1 billion in the United States annually [26]. They are also agricultural pests in oil palm plantations in Malaysia, sugarcanes in Pakistan, and maize, which is a staple food in Africa [4,6,71,81,84].

The potential use of *M. anisopliae* as an effective biological control of termites has been documented in previous studies. The efficacy of *M. anisopliae* is affected by three factors: the strain of *M. anisopliae* used, the dosage of *M. anisopliae* conidia, and the genera and species of termites which have a different susceptibility to the same strain of *M. anisopliae.* The mortality of termites is dose dependent, whereby increasing the dosage of conidia of *M*. *anisopliae* increases the mortality of termites. Specifically, the highest insecticidal dosage of conidia is approximately 1 × 10^6^ conidia/mL to 1 × 10^10^ conidia/mL [4,6,84,85,86]. The least number of days taken to reach 100% was between three days and 21 days post-inoculation. 

In different studies, different strains of *M. anisopliae* and different hosts are used, which do not allow for a consistent comparison for improvement. The non-uniformity stems from the strains of *M. anisopliae* being localised to their respective regions and the termites that caused the most damage are different in different regions and countries. Besides, the same strain of *M. anisopliae* could have a different virulence towards different genera of termites [6]. One of the primary challenges is finding an effective way to overcome the defensive behaviour of termites, such as the repellency of termites towards conidia, allogrooming to remove conidia from infected termites, alarm behaviour, the walling off of infected colonies, avoiding or burying infected cadavers, the immune response by producing antimicrobial compounds, and the activation of a cellular and humoral response [2,4,68,87]. These defense mechanisms prevent the transmission of conidia from one infected termite to another, which is crucial for causing an epizootic among termite populations. Current methods are dependent on the application of a high amount of conidia on termites which could be removed by other termites via allogrooming, leading to the inefficiency of the conidia as a biological control. 

The repellency of termites, which leads to the allogrooming behavior, was demonstrated to be due to the ability of termites to detect the odour of conidia [88]. The more virulent the conidia, the stronger the odour, and the more repellence displayed by the termites [68,89,90]. It was found that the antennal olfactory receptors of worker termites were able to detect the volatile compounds produced by *M. anisopliae*, which are mainly composed ofparaffins such as *N*-tetradecane [88]. The allogrooming behavior of termites to *M. anisopliae* was a contradictory issue as Chouvenc et al. [91] and Yanagawa and Shimizu [2] hypothesised that allogrooming prevents the spread of conidia in the population due to conidia being ingested by groomers, while Balachander et al. [92] hypothesised that allogrooming allows the spread of conidia as the mouth of the grooming termite most likely contaminated, which allows it to transmit the conidia to other termites. Hussain et al. [87] proposed that whether allogrooming spreads conidia depends on the percentage of infected individuals in the colony. Therefore, to determine whether allogrooming behavior promotes or prevents the spread of conidia, sampling of the termite population in the field could be conducted to determine the density of the termite population before and after conidia application compared to a control termite population. A reduction in the termite population could indicate the possibility of death of termites due to conidial infection. 

To reduce the repellence displayed by termites, studies have focused on two aspects: the reduction of repellency by termites and the nest embedded sensor in the termite nest. Chouvenc et al. [91] employed bait and direct application with attractants such as cardboard powder or cellulose to reduce the repellence of the conidia which could not only kill termites, but spread the conidia among healthy termites in the population. 

Another study by Bulmer et al. [93] demonstrated that by targeting the Gram-negative bacterial binding proteins present in the nest of termites which function as a nest-embedded sensor that removes pathogenic compounds, the termites are more susceptible to the effects of *M. anisopliae.*

The drawback in the studies is that in field experiments, it is uncertain whether *M. anisopliae* cause the mortality of termites or repel the termites such as *C. formosanus*. This has a significant impact because if *M. anisopliae* were to only repel termites, *M. anisopliae* would not be effective in completely eliminating termites, especially in buildings such as temples and historic monuments, and the conidia would have to be consistently reapplied [6].

Chovenc et al. [91] reported that *M. anisopliae* strains with the ability to form appressorium more quickly are more virulent and able to cause higher mortality. To achieve this, genetic manipulation could be implemented by increasing the expression of genes which are responsible for appressorium formation or conidia formation to increase the virulence of *M. anisopliae*. It was also found that 90% of the conidia that were ingested by groomers could not germinate in the gut of termites, although conidia were found to attach to the lining [94]. Zhang et al. [67] inserted a gene from *Bacillus thuringiensis*, which encodes for the insect midgut-specific toxin Vip3Aal, into *M. anisopliae*. The genetically modified *M. anisopliae* was found to successfully infect a host that is usually resistant to *M. anisopliae* by the ingestion of conidia instead of penetration of the cuticle. Hence, the addition of a specific gene which is toxic in the midgut of termites could be the key to a more virulent *M. anisopliae*. Further studies should be done on the fungistatic compounds present in the gut microbiota of termites to determine which midgut toxin is resistant to the fungistatic compounds in the termites [2].

### 7.2. Biological Control of Mosquitoes

#### 7.2.1. Adult Mosquitoes

At the adult stage, all previous studies agreed that *M. anisopliae* was able to cause a significantly higher mortality in *Anopheles* spp. and *Aedes* spp. compared to moquitoes which are not treated. For *Anopheles* spp., studies found that more than 90% of *Anopheles* spp. exposed to *M. anisopliae* were dead 14 days post-inoculation. This means that *Anopheles* spp. can be prevented from transmitting malaria since the *Plasmodium* parasite required approximately 12 to 14 days to develop in the mosquito into infectious sporozoites, at which point the mosquito could transmit malaria [66]. 

Paula et al. [9] and Mnyone [74] found that blood fed mosquitoes were less susceptible to *M. anisopliae* compared to mosquitoes which did not have a blood meal and mosquitoes which were fed with sucrose. Blood fed mosquitoes were suggested to have more nutrient reserves, resulting in fungus requiring more time to deplete the nutrients in the host before causing mortality of the adult mosquitoes. They also suggested that blood-fed mosquitoes may have a stronger immune system to overcome the infection by *M. anisopliae*. The reduced susceptibility of *Aedes* spp. was found to persist for approximately 96 hours to 120 h where the susceptibility was no different from the controls after that period [9].

Reyes-Villaneuva et al. [95] found that male mosquitoes which were contaminated with conidia were also able to infect female mosquitoes during mating. Male mosquitoes which were contaminated with the fungus were found to have a lower sperm production and the time invested in copulation was longer. This means that the fungus affects the sexual behaviour of the male mosquitoes and further studies need to be conducted on how the fungus actually alters the behaviour. However, the ability of the male mosquitoes to find and inseminate females is not affected and this signifies that there is still potential for the conidia of the fungus to be transmitted from one mosquito to another in a population [95]. The fecundity of females suffered a reduction after being infected with *M. anisopliae*.

#### 7.2.2. Mosquito Larvae and Eggs

Mosquitoes such as *Aedes aegypti* and *Aedes albopcitus* are responsible for dengue epidemics, dengue haemorraghic fever, chikunguya, and yellow fever. Fifty million people are estimated by the World Health Organization to be infected with dengue fever worldwide every year [9,96]. Dengue is transmitted by *A. aegypti* in both tropical and subtropical regions in the world [60,72]. *Anopheles* spp. are vectors responsible for the transmission of malaria, while *Culex quinquefasciatus* transmits *Bancroftian filariasis* which is responsible for causing filariasis in 25 million people in India alone [16,61,79].

For the biological control of vectors of human diseases, especially *Aedes* spp. and *Anopheles* spp., studies have been conducted that investigate the effects of *M. anisopliae* on the three different developmental stages of mosquitoes which are the egg, larvae, and the adult stage. For all three stages, *M. anisopliae* was found to have insecticidal activity and is a potential entomopathogenic fungus. There are some factors which need to be taken into consideration when targeting all three developmental stages, but there are also other factors which only apply to a particular developmental stage. 

At the larval stage, Bukhari et al. [97] found that conidia in ShellSol T formulation cause a mortality rate in larvae that was four times higher than the control. This may be due to its ability to spread the conidia more uniformly on the water surface. The spores did not clump together, which could reduce the amount of free conidia available to infect the host. The conidia in the ShellSol T formulation also caused significantly higher larval mortality seven days after application compared to unformulated conidia or conidia formulated in WaterSavr. This formulation, however, may only be effective for targeting the larvae of *Anopheles* spp. and *Culex* spp., but not *Aedes* spp. This is because the primary feeding sites of *Anopheles* spp. and *Culex* spp. are on the water surface, but the primary mode of feeding of *Aedes* spp. is through browsing and not on the water surface [18,80]. Greenfield et al. [18] found that “dry” conidia were more virulent than “wet” conidia, contradicting the results of Bukhari et al. [97]. The “wet” conidia were formulated in 0.03% to 0.1% aqueous Tween 80 and conidia formulated in 0.1% Tween 80 sank instead of spreading uniformly on the water surface. The difference in results could also be attributed to the location of the experiment conducted by Greenfield et al. [10] which was in the laboratory, while the experiments conducted by Kalamakannan and Murugan [16] and Bukhari et al. [97] were conducted in the field and were exposed to environmental conditions. The oil formulated conidia were predicted to be more persistent and virulent because they display a better protection against UV radiation, fluctuating temperature, and humidity, which may decrease their viability and subsequently, their virulence [18,97]. For the adult stage, it was found that mineral oil formulation had no repellence against adult mosquitoes [98]. In addition, Carolino et al. [98] found that conidia formulated in a combination of vegetable oil and paraffin oil were able to maintain the viability and virulence of conidia of *M. anisopliae* for 18 to 23 days.

For relative humidity, it was found that the ovicidal activity of *M. anisopliae* is the greatest when the mosquito eggs treated with conidia are exposed to 98% relative humidity for at least 10 days [64,72,96]. The higher the humidity that the fungus contaminated eggs are exposed to, the greater the amount of mycelium and new conidia formed on the treated eggs. The lowest cumulative eclosion was found when eggs were incubated with 98% relative humidity. At a humidity below 86%, no mycelium or conidia were found on the fungus treated eggs. However, since these conditions can only occur during rainy seasons or subterranean areas where female mosquitoes oviposit throughout the year, their experiments also tested alternating humidities, which show that the eclosion of larvae decreases when the eggs treated with fungi are exposed to greater than 98% relative humidity or by increasing the days of exposure of eggs to relative humidity. Despite this, the lack of eclosion does not necessarily mean that the larvae inside the eggs had died [79,96]. Future research should be conducted to determine whether the larvae in the eggs which did not eclose were dead or alive after having been treated with the conidia of *M. anisopliae*. Luz et al. [99] also found that the incubation of eggs in leaf litter for two weeks showed the presence of abundant mycelium and new conidia on the treated eggs with no larvae observed after two weeks, while treated eggs in soil showed little or no presence of fungi and a 10% eclosion of larvae. Hence, it was suggested that the higher water content in leaf litter compared to soil reduced the growth of the survival of eggs due to high humidity.

The mortality of larvae at different stages was dose-dependent, where increasing the dosage of applied conidia was found to increase the larval mortality. Younger instar larvae (L2) were found to be more vulnerable to *M. anisopliae* compared to older instar larvae (L4) and adult mosquitoes [18,19]. However, Greenfield et al. [10] found that early and late instar larvae of *Anopheles* spp., *Culex* spp., and *Aedes* spp. are equally susceptible. 

However, the challenges of using *M. anisopliae* are its slow mode of action, inconsistent results compared to chemical insecticides, and the fact that it only targets the larval population which are present. *M. anisopliae* has to be reapplied after the site has been reinhabited by larvae [40,80,100]. The slow mode of action of *M. anisopliae*, which usually takes seven to 14 days to reach >90% mortality, does not fulfil the requirement to be a vector control product by World Health Organization Pesticide Evaluation Scheme (WHOPES) [34]. One of the gaps in these studies is that most of the studies are conducted in a laboratory condition where the eggs of the mosquito are treated with fungus. The time taken between the laying and eclosion of larvae from the eggs also needs to be considered because the application of the conidia, which is only virulent for a certain period of time, must coincide with the most susceptible stage of the mosquitoes for the maximum efficacy. Further studies should also be conducted on whether treating the eggs with fungus or treating the possible breeding sites of the mosquitoes with fungus before they lay their eggs may be a more practical approach as the conidia of fungus are susceptible to UV radiation and environmental conditions. The mechanism of infection of *M. anisopliae* on the mosquito eggs and how high humidity reduces larval eclosion should be further studied.

Further studies could be conducted on the formulation of conidia which could best protect the conidia from environmental conditions that could inactivate conidia to maintain the virulence and which type of oil could repel mosquitoes. The effects of *M. anisopliae* on non-target organisms and the environment should also be studied in order to determine the safety of the application of *M. anisopliae* as a biological control agent.

### 7.3. Biological Control of Ticks

The efficacy of *M. anisopliae* in killing the nymphs and adults of many genus and species of ticks has been demonstrated in many studies for *Rhipicephalus microplus*, *Rhipicephalus sanguineus*, *Haemaphysalis qinghaiensis*, *Hyalomma excavatum*, and Ixodes ricinus [25,28,76,78,101,102,103,104,105]. Although studies have shown that the conidia of *M. anisopliae* reduced the percentage of egg production and hatchability, the precise mechanism of how *M. anisopliae* caused a decline in egg production and hatchability is still unknown [105,106]. Camargo et al. [25] found that the mortality of the larvae of *R. miroplus* is dose dependent, where mortality increases with an increasing concentration of conidia.

There are currently two types of application of *M. anisopliae* which are the application of the fungus parasitic stage onto cattle ticks and the non-parasitic stage onto tick larvae in vegetation [107]. Further studies needed to be consider whether the application of conidia onto cattle or onto vegetation would be more effective. This is because it was found that the ability of a fungal biopesticide containing *M. anisopliae* to kill the ticks on the cattle was affected. Leemon et al. [97] suggested that the higher temperature on the surface of the skin of the cattle may be responsible for the lower efficacy of *M. anisopliae*.

Quinelato et al. [101] also found that different isolates have a different virulence towards the same host. They hypothesised that this may be due to their genetic variability and not their origin. Besides, the larval developmental stage was found to be the most susceptible to *M. anisopliae*, followed by nymphs and adults [8,104,105]. The reasons why larvae are more susceptible, however, have not yet been studied and more research should be done. Leemon et al. [80] and Camargo et al. [25] cited from previous studies that the difference in cattle breeds can also result in the different efficacy of *M. anisopliae* on the mortality of cattle ticks. 

## 8. *Metarhizum acridum* as a Biological Control of Locusts and Grasshoppers

Locusts and grasshoppers are major agricultural pests which have caused huge agricultural and economic losses. In Africa, locusts and grasshoppers cause damages on crops such as maize, beans, and sweet potatoes. Approximately USD 400 million was spent to control these agricultural pests, besides providing food and assistance to affected communities [5]. Extensive research on the usage of *M. acridum* on locusts and grasshoppers has been conducted by the LUBILOSA program (Lutte Biologiquw contre les Locustes et les Sauteriaux) in Africa and CSIRO (Commonwealth Scientific and Industrial Research Organisation) in Australia over the last 10 years [1]. *M. acridum* is a specialist, having a narrow host range where it infects only locusts and grasshoppers [22].

Previous studies have found that *M. acridum* has an insecticidal effect against locusts and grasshoppers, which are the main agricultural pests. *M. acridum* is currently used as a biological control agent in Mexico, Africa, and Australia for the control of locusts and grasshoppers [5,7,108]. Commercial biopesticides using *M. acridum* as an active ingredient have been developed in both Africa and Australia, known as GreenMuscle^®^ and GreenGuard^®^, respectively, to act as control agents against locusts and grasshoppers [7,108]. GreenMuscle^®^ has been recommended by FAO and has obtained registration for its usage. However, few people have used GreenMuscle^®^ due to its slow mode of infection, high costs, temperature dependency, and the instability of the formulation developed [108].

Factors that affect the efficacy of *M. acridum* are the timing of the application of conidia, the temperature during the infection of *M. acridum*, the strain of the *M. acridum* used, the medium that is used to culture *M. acridum*, and the method of application of the conidia [5,7,12,83,108].

For the timing factor, it was found that younger nymphal instar of locusts are more susceptible than older nymphal instars. It was observed that 10^10^ spores mL^−1^ caused higher mortality for 3rd nymphal instars compared to 5th nymphal instars 15 days post-inoculation in 2005, but not in 2006 [108]. Klass et al. [109] also suggested that for the successful control of locusts, it is better to target early nymphal instars to prevent later nymphal instars from developing into adults which could reproduce and expand the locust population. Further studies should focus on why younger nymphal instars are more susceptible to the effects of conidia, whether the environmental conditions influence its susceptibility, and how increasing the conidia dosage could cause higher mortality.

Studies have found that the suitable temperature range for the germination of the conidia of *M. acridum* was from 28 to 33 °C with 28 °C, which demonstrated the highest germination rate of conidia, longest mycelium, and the highest conidia production. This perhaps signifies that the virulence of *M. acridum* is the highest at 28 °C since the virulence of *M. acridum* increases with higher conidia germination, enabling a higher growth of mycelium [83]. Many studies reported that locusts which have the ability to thermoregulate have a higher tendency to bask under the sun after being infected by *M. acridum*. The act of basking under the sun elevates their body temperature to around 35 °C and the temperature can rise up to 37 to 40 °C during summer. Temperatures above 35 °C could inactivate the conidia of *M. acridum*, inhibiting their development and subsequently reducing the mortality of the host [1,110].

Different strains of *M. acridum* contribute to the difference in their virulence and may be due to the differences in their genetic composition, resulting in the differences in their virulence and pathogenicity [5]. This may also be due to the different climates in different geographical regions where the temperature and length of daylight hours may vary. Regions with longer daylight hours and a higher temperature may require more virulent *M. acridum* as locusts could elevate their body temperature for longer periods, while regions with longer and cooler nights require a lower dosage of conidia to result in a higher mortality of locusts [109].

Guerrero-Guerra et al. [7] found that the viability of conidia sprayed onto soil is much longer, with a figure of one year and four months compared to the conidia sprayed onto the vegetation with a figure of eight months. For the vegetation cover, it was found that the density of the vegetation cover does not affect the efficacy of *M. acridum.* The number of *M. acridum* conidia on the soil was reported to be negatively correlated with relative humidity and positively correlated with wind velocity. Further studies should be done on the best application method and the minimum dosage of conidia needed to be applied to a particular area for highest mortality [7].

One of the main challenges is to overcome the behavioural fever of locusts, which could inactivate the conidia of *M. acridum*. The inactivation of the conidia of *M. acridum* could reduce the efficacy of *M. acridum*, leading to a decreased mortality of locusts. Genes that are heat tolerant could be genetically engineered into *M. acridum* to overcome this challenge. The second challenge is to identify the best formulation and application method of conidia in the field to ensure the viability and persistence of conidia due to environmental conditions such as rain, humidity, and solar radiation. The timing of application is also vital to target early nymphal instars to reduce the number of adult locusts which could reproduce, thus expanding the population of locusts. The third challenge is finding a suitable strain of *M. acridum* for a particular geographical location due to different environmental conditions in different regions which can influence the thermoregulatory behaviour of locusts, subsequently affecting the efficacy of the conidia of *M. acridum*. The fourth challenge is maintaining the residual effect of *M. acridum*. To maintain the residual effect, the exact mode of locusts being infected in the field has to be first understood. Atheimine et al. [14] suggested that the three modes of locust being infected by the conidia are via direct contact with the conidia of *M. acridum*, secondary pick up of conidia from treated vegetation, and horizontal transmission from cadaver infected with conidia. [13]

## 9. Conclusions

The general mode of infection of *Metarhizium* fungus can be categorised into adhesion, germination, appressorium formation, penetration, extrusion, and sporulation [42,56,111].

*M. anisopliae*, which is a generalist, is a potential biocontrol agent of termites, mosquitoes, and cattle ticks, while *M. acridum* is a potential biocontrol agent of locusts and grasshoppers. However, many challenges, such as the slow mode of action and unpredictable results of the application of these fungi, have to be overcome to maximise the efficacy of both *M. anisopliae* and *M. acridum*. Studies have been conducted to speed up the mode of action by genetically engineering both *M. anisopliae* and *M. acridum*. However, the efficacy of these genetically engineered fungi in the field has not been published. The effects of these fungi on the effects of non-target organisms and the environment should also be considered before using them in the field.
